# On the Use of Nitrate of Silver in Testitis and Carbuncle

**Published:** 1873-11

**Authors:** 


### ON THE USE OF NITRATE OF SILVER IN TESTITIS AND CARBUNCLE.

The uniform success in my hands, during the last five years of the use of the solid nitrate of silver, in cases of testitis and anthrax has led me to recommend the more general adoption of this mode of treatment in these cases, as I am sure it requires only that its eminently satisfactory results should be known.

And first as to testitis. The ordinary commencement of the treatment of swelled testicle in the acute form is still too frequently the application of leeches. Of late years the plan of puncturing with a thin, sharp knife, the tunica albuginea of the hard and painful testicle, as recommended by Mr. Henry Smith, has been tried by many surgeons, and with certainly, generally favorable results. The former mode of treatment I have long given up; the latter I have been willing and anxious to try; but so favorable and prompt has been the effect of the application of nitrate of silver, that I have not once had an opportunity of doing so.

The plan I adopt is the following: The scrotum is held in such way that the portion of it which surrounds the swollen testicle is rendered—if not already so—sufficiently tense to present a tolerably smooth surface of skin. This is first wetted by means of a sponge, or better, by a piece of lint previously dipped in water, and the solid nitrate of silver is then carefully and equally applied over the whole testicle. A suspensory bandage and rest are of course prescribed, and such general treatment as may be required. Pain disappears in from *two* to *six* hours, and this is accompanied and followed by a gradual diminution of the swelling, the reduction being generally about one-third during the first three days. Considerable smarting occurs for a short time after the application, and sometimes there is vesication. The further treatment of the case becomes exceedingly simple.

During the last five years I have treated in this way a large number of cases, and only twice has the application failed to reduce both pain and swelling; in both of these the appearance of the skin of the scrotum showed that the remedy had been but partially applied, and in both the symptoms were removed by a second and more careful application of the caustic. The rapid effect of this treatment is still more marked in cases of double testitis, the whole skin and dartos of the scrotum contracts firmly around the testes, speedily relieving the engorgement of the capillaries and seeming to produce a uniform gentle pressure on the swollen organs. I have never known abscess to occur in any case treated with nitrate of silver.

In both the forms of anthrax—carbuncle and boil—the application of nitrate of silver affords the most speedy means of cure. One looks back with horror, at the heroic plan of treating carbuncles, sometimes enormous in their size, by crucial incisions; cases, too, occur to one's memory in which, in spite of this operative procedure, the carbuncle still went on increasing in size; where in fact, the incisions not only did no good, but positively did harm, by the shock to the patient and the increased risk of pyæmia. A lecture upon this subject by Sir James Paget, appeared in the *Lancet*, Jan. 16th, 1869, in which he strongly coudemned this mode of treatment.

The treatment he recommends is at first a piece of emplastrum plumbi with a hole in the centre; then resin cerate on lint, covered over with a large poultice (half linseed and half bread); and other deodorizing fluid. With these measures must, of course, be combined cleanliness, fresh air, and a careful regulation of diet.

I have found, however, that the duration of carbuncle is very materially diminished and its extension cut short, by preceding this treatment by the application of nitrate of silver freely over its surface, repeated if necessary, once or twice after intervals of two days. Immediately after the application, a small soft pad of dry lint is applied and retained by means of a piece of strapping and a bandage. The after treatment is the same as Sir James Paget recommends, except that the poultice will be unnecessary, and the internal administration of iron or other tonic will generally be found useful.

Boils are treated in the same way, and will seldom require a second application of the caustic.

The *modus operandi* of the application of nitrate of silver in these cases seem to be the energetic stimulation and consequent contraction of the capillaries and small arteries of the part, whereby engorgement is diminished, the vessels are placed in a condition for returning to a healthy function, and morbid exudation is diminished, arrested and removed.—*London Practitioner.*

